# SGRT‐based stereotactic body radiotherapy for lung cancer setup accuracy and margin of the PTV

**DOI:** 10.1002/acm2.14195

**Published:** 2023-11-01

**Authors:** Hai‐Liang Guo, Wei‐Wei Wu, Yan Huan, Huai‐Wen Zhang

**Affiliations:** ^1^ Department of Oncology the First Affiliated Hospital of Gannan Medical University Ganzhou China; ^2^ Department of Radiotherapy the Affiliated Cancer Hospital of Gannan Medical University, GanZhou Cancer Hospital Ganzhou China; ^3^ Department of Oncology People's Hospital of Qianxinan Buyi and Miao Minority Autonomous Prefecture Qian xinan China; ^4^ Department of Radiotherapy Jiangxi Cancer Hospital The Second Affiliated Hospital of Nanchang Medical College, NHC Key Laboratory of Personalized Diagnosis and Treatment of Nasopharyngeal Carcinoma Nanchang China; ^5^ Department of Oncology The Third People's Hospital of Jingdezhen Jingdezhen China

**Keywords:** intrafraction error, lung cancer, stereotactic radiotherapy, surface‐guided radiation therapy

## Abstract

**Objective:**

Surface‐guided radiation therapy (SGRT, AlignRT) was used to analyze motion during stereotactic body radiotherapy (SBRT) in lung cancer patients and to explore the margin of the planning target volume (PTV).

**Methods:**

The residual errors of the AlignRT were evaluated based on grayscale cone‐beam computed tomography registration results before each treatment. AlignRT log file was used to analyze the correlation between the frequency and longest duration of errors larger than 2 mm and lasting longer than 2 s and maximum error with age and treatment duration. The displacement value at the end of treatment, the average displacement value, and the 95% probability density displacement interval were defined as intrafraction errors, and PTV1, PTV2, PTV3 were calculated by Van Herk formula or Z score analysis. Organ dosimetric differences were compared after the experience‐based margin was replaced with PTV3.

**Results:**

The interfraction residual errors were Vrt_0_, 0.06 ± 0.18 cm; Lng_0_, ‐0.03 ± 0.19 cm; Lat_0_, 0.02 ± 0.15 cm; Pitch_0_, 0.23 ± 0.7°; Roll_0_, 0.1 ± 0.69°; Rtn_0_, ‐0.02 ± 0.79°. The frequency, longest duration and maximum error in vertical direction were correlated with treatment duration (*r* = 0.404, 0.353, 0.283, *p* < 0.05, respectively). In the longitudinal direction, the frequency was correlated with age and treatment duration (*r* = 0.376, 0.283, *p* < 0.05, respectively), maximum error was correlated with age (*r* = 0.4, *P* < 0.05). Vertical, longitudinal, lateral margins of PTV1, PTV2, PTV3 were 2 mm, 4 mm, 2 mm; 2 mm, 2 mm, 2 mm, 3 mm, 5 mm, 3 mm, respectively. After replacing the original PTV, mean lung dose (MLD), 2‐cm^3^ chest wall dose (CD), lung V_20_ decreased by 0.2 Gy, 2.1 Gy, 0.5%, respectively (*p* < 0.05).

**Conclusion:**

AlignRT can be used for interfraction setup and monitoring intrafraction motion. It is more reasonable to use upper and lower limits of the 95% probability density interval as an intrafraction error.

## 1 INTRODUCTION

Stereotactic body radiotherapy (SBRT) is effective in the treatment of non‐small cell lung cancer.^1^, ^2^, ^3^ Due to the high dose per fraction, the patient needs to be scanned by cone‐beam computed tomography (CBCT) before each procedure to ensure the accuracy of the irradiation position^4^ and to control the intrafractional patient shift and motion during treatment delivery. However, CBCT can only correct the interfraction setup error and cannot monitor the changes in patient position in real‐time during execution of the plan. Surface‐guided radiotherapy (SGRT) has been demonstrated to have high positioning accuracy in the detection of phantoms and patient surfaces,^5^, ^6^, ^7^, ^8^ which can be used to monitor the position of patients in real‐time to assess translation and rotation, without additional radiation risk. Therefore, it is usually used together with CBCT for patient setup verification and displacement monitoring.^9^, ^10^, ^11^

Previously, the intrafraction motion was obtained by performing CBCT after correcting the tumor dislocation before treatment,^12^ and the minimum safe boundary was calculated using the Van Herk formula to ensure adequate dose coverage. However, the application of SBRT in the treatment of lung cancer usually takes a relatively long time, which is easy to cause easily causes discomfort and anxiety in patients, leading to involuntary movement, and thus increasing the number of tumor movements in patients. The shift detected after whole treatment or after single irradiation field treatment completed may not represent the change of the patient's position during the whole treatment process, which may overestimate or underestimate the margin of the tumor. Multiple CBCT scans will significantly increase the absorbed dose of patients. However, whether this will lead to secondary low‐dose cancer remains to be confirmed by further research.

Many studies have found the results of SGRT and CBCT are in good agreement, which can improve the radiation protection of patients and save treatment time, so as to monitor the patients' fractional movement without additional CBCT. Heinzerling et al.^13^ compared the average shift detected by SGRT to the resulting shift on subsequent CBCT during treatment, and no significant difference was found between the two. Nguyen et al.^14^ used CBCT midway through breath‐hold lung SBRT treatment and found that the values from an AlignRT correlated with CBCT shifts in the longitudinal and lateral directions. The mean difference between values and CBCT shifts remained <0.03 mm in the vertical direction and <0.4 mm in the other five directions, and the tumor remained within the planning target volume (PTV) whenever the surface position was within SGRT tolerance. Previous studies have reported similar findings.^15^, ^16^ Sarudis et al.^17^ demonstrated that SGRT is a viable approach for lung SBRT. Many studies have found the results of SGRT and CBCT are in good agreement, it can improve the radiation protection of patients and save treatment time, so as to monitor the patients' fractional movement without additional CBCT.

When SGRT is used to continuously monitor patients during treatment, it may be feasible to reduce the PTV margin of SBRT in the lung,^13^ but as far as we know, we have not seen other papers use SGRT to determine this reduction. The AlignRT system (vision RT, UK) reconstructs the patient's body surface through near‐infrared imaging, monitors and judges the changes of the patient's body surface through the registration algorithm, and records the displacement information of the body surface in a text file in real‐time. In this study, the three‐dimensional displacement of patients with non‐small cell lung cancer during SBRT treatment was studied by offline analysis of the treatment logs, so as to provide a scientific reference for the edge of tumor target.

## 2 MATERIALS AND METHODS

### 2.1 General patient information

From January 2021 to December 2021, 12 lung cancer patients who underwent SBRT were selected, including nine males and three females, with a median age of 68 years (43‐79 years). The median tumor volume was 30.54 cm^3^ (11.46‐89.22 cm^3^). Among them, there were eight cases of the peripheral type and four cases of the central type, with single tumors in all cases. Eight patients were treated end‐expiratory respiratory cycle phases, and four patients were treated whole respiratory cycle. The prescribed dose was 45−56 Gy, and the number of treatment fractions was 3−8; all treatments were administered every other day. No patients had contraindications to radiotherapy. All patients provided written informed consent for radiotherapy before treatment.

### 2.2 CT scan and plan design

All patients were fixed with a stereotaxic frame (Model R62400) and vacuum negative‐pressure pads (Klarity Medical & Equipment, China), and a marker was established to affix lead pellets on a stable area of the chest skin that was less affected by breathing movements and arm pulling. 4D CT scanning of the chest was performed using a 16‐slice CT system (Discovery RT, GE) based on free breathing using the following parameters: scanning voltage, 120 kV; current, 215 mAs; and slice thickness, 2.5 mm. The 4D CT images were reconstructed using phase binning with 10 different phases of the breathing cycle and transmitted to the Eclipse 15.5 treatment planning system (VARIAN, USA). The gross tumor volume (GTV) (including respiratory movement) was delineated as the tumor tissue seen through the lung window setting in the maximum intensity projection (MIP) of the whole respiratory cycle or end‐expiratory respiratory cycle phases, and the internal target volume (ITV) was set equal to the GTV. The PTV was created around the ITV using 5 mm symmetrical edges according to previous experience.^18^, ^19^ For all patients, a VitalBeam accelerator (VARIAN) was used for plan design, with a Millennium 120‐leaf MLC, 6 MV flattening filter‐free (FFF) X‐ray radiation, and a 1400‐monitor‐unit/min dose rate. The isocenter of the radiation field was set in the center of the tumor target area. Using the Acuros XB algorithm, the 3D dose was calculated with a grid size of 1.25 mm. The aperture shape controller was set to very high to reduce planning complexity and monitor units, and dual‐arc volumetric modulated arc radiotherapy (VMAT) was used to improve execution efficiency. According to the lung SBRT 0813 report of the Radiotherapy Oncology Group (RTOG) and the SBRT expert consensus on early non‐small cell lung cancer in China (2019),^20^, ^21^, ^22^ all planned prescriptions, gradient indexes and major organ limits were determined.

### 2.3 Pretreatment setup and plan execution

**FIGURE 1:**
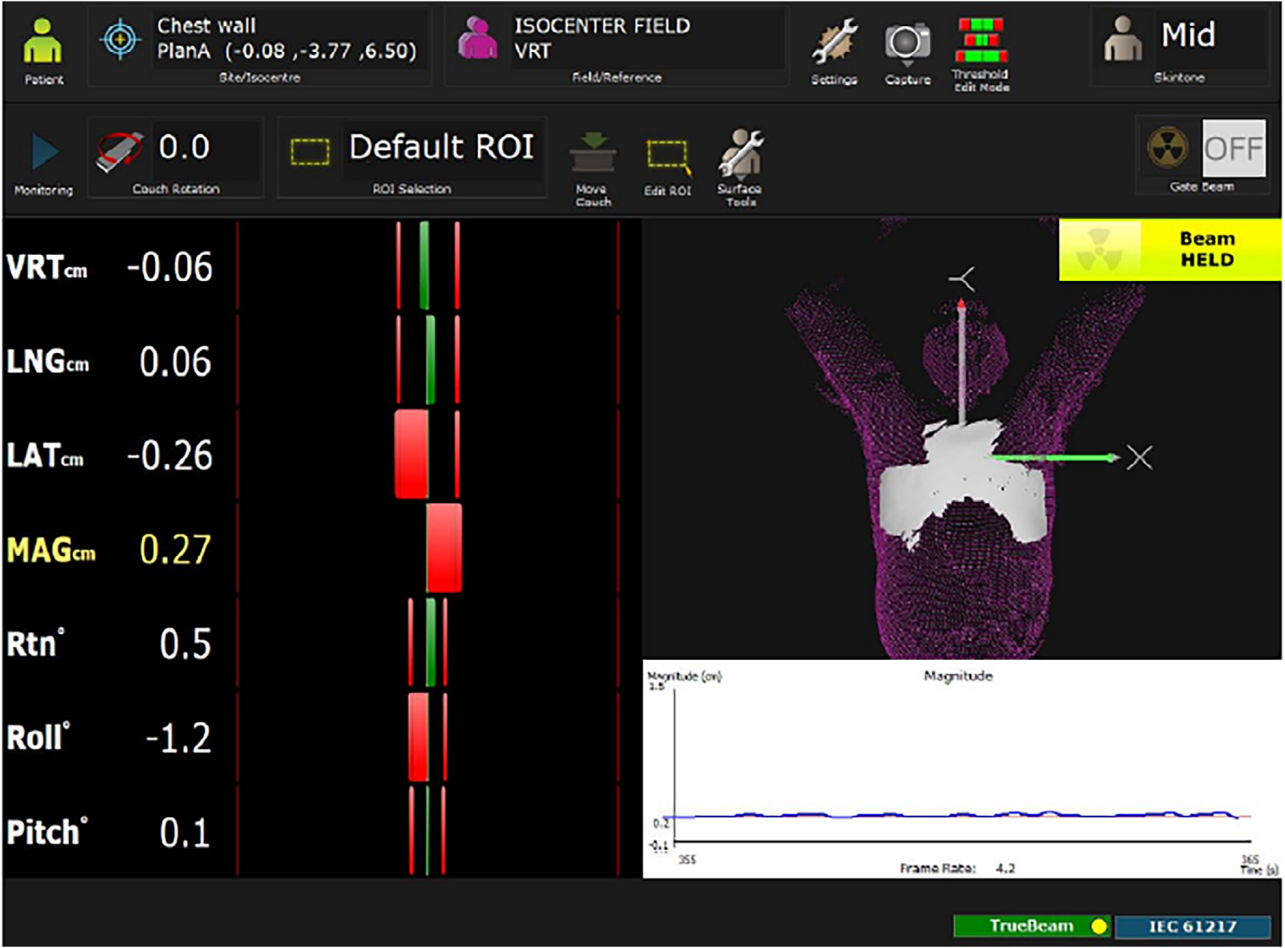
Software interface of the AlignRT system.

A senior radiotherapy physicist transferred the RT structure and RT plan files to the AlignRT system. After importing the files, the radiotherapist outlined an inverted‐T‐shaped symmetrical surface with the sternum as the center that was not changed by the patient's breathing motion as the infrared tracking area of interest (Figure 1). After switching to the AlignRT treatment mode, three cameras in the treatment room obtained the patient's body surface data. The error in six dimensions was first compared with the delineated reference surface. Then, the gantry was rotated to ensure that it would not block the cameras during the treatment process, which could cause monitoring data errors. Real‐time position management (RPM, Varian) was used to monitor the respiratory state of the patient, judge whether the trigger threshold had been reached, and turn the beam on or off.

**FIGURE 2:**
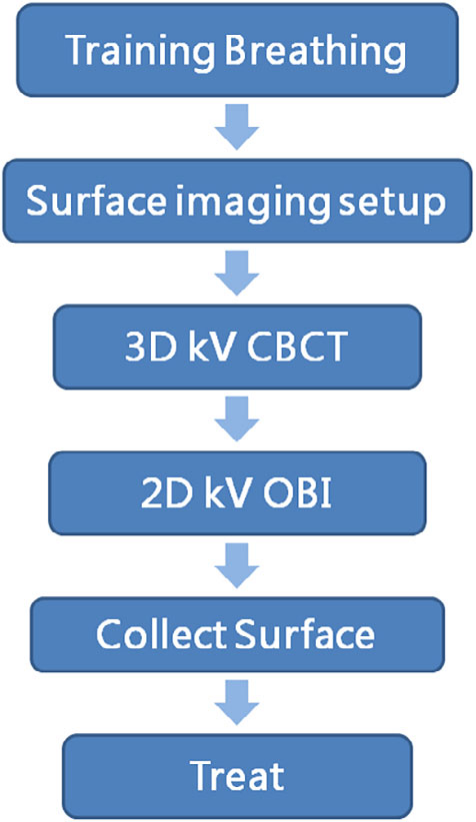
Treatment process.

The AlignRT system collected and compared the patient's body surface data at a frequency of 0.12 s and recorded the displacement in six dimensions in a delta text log file written in real time.^23^ The patient treatment process is shown in Figure 2.

### 2.4 Correlation analysis

Grayscale CBCT registration was performed before the start of each treatment, and the results were used to evaluate the residual errors of the interfraction setup by AlignRT in six dimensions: vertical (Vrt_0_), longitudinal (Lng_0_), lateral (Lat_0_), couch rotation (Rtn_0_), couch tilt (Pitch_0_), and couch roll (Roll_0_). We referred to the threshold of unacceptable patient position shift in fractional treatment with a high dose rate proposed by Heinzerling et al.,^12^ that is, displacement in any of the vertical (VRT), longitudinal (LNG) or lateral (LAT) dimensions greater than 2 mm and lasting longer than 2 s. We retrospectively analyzed each patient's treatment log files using Microsoft Excel 2003 and analyzed the correlation of the frequency of occurrence (Frequency), the longest duration (Max Time), and the absolute value of the maximum error detected in the whole fractional treatment (Max Error) with the age of the patient (age) and single treatment time (treatment time).

### 2.5 PTV margin analysis

In this study, we recorded three sets of data to describe and define the intrafraction error and calculate the respective PTV margin. First, the intrafraction error was defined as the patient displacement at the end of treatment relative to the start of treatment. The displacement of the patient's body surface at the end of each treatment recorded by the AlignRT system was taken as intrafraction error 1. The mean value of each setup error of each patient was taken as the individual systematic error, and the standard deviation of the setup error was taken as the individual random error. The group systematic error (Σ) is expressed by the standard deviation of the individual systematic error, and the group random error (ϕ) is expressed by the standard deviation of the individual random error.^24^ According to the study by van Herk et al., an 80% isodose line covering 90% of the CTV of patients in the less fractionated SBRT plan was calculated to obtain the minimum safe margin, that is, PTV1:

(1) $$M\;=\;2.5\tau +0.84\sqrt{\sigma^{2}+\sigma_{P}^{2}}-0.84\sigma_{P}$$

(2) $$\mathrm{of}\;\mathrm{which}\;\;\tau =\sqrt{\Sigma^{2}+\varphi^{2}/n},$$

where $\sigma_{p}$ = 6.4 mm is the penumbra value of the X‐ray beam in the lung tissue, and *n* = 12 is the number of sample cases.^25^, ^26^, ^27^, ^28^ Second, we took the average displacement of the patient in each treatment recorded by the AlignRT system as intrafraction error 2 and applied Formula (1) and Formula (2) to calculate the three‐dimensional parameters and obtain the margin of PTV2. We believe that less than 5% probability indicates a low probability for an event; thus, we used the Z score analysis method to calculate the 95% probability density shift interval for each treatment in the log file (μ−2s,μ+2s) as intrafraction error 3, where μ is the mean and s is the standard deviation. The shift interval data of each patient and each treatment were combined into the population data, from which a histogram was drawn to evaluate the normality of the distribution. The 90% probability density interval (μ−1.65s,μ+1.65s) of the population data was calculated to determine the minimum intrafraction margin range required for each anatomical layer of the population to have a 90% probability of achieving PTV3.^29^

To verify the rationality of the margin of the three PTVs, the margin value in the three directions was compared with the 95% probability density shift interval value recorded in the log file. When the PTV was greater than or equal to the interval, the tumor was considered covered by the PTV; otherwise, it was considered missed. The proportion of tumors covered by the PTV was calculated for the entire group for all treatment times. The most reasonable margin of PTV (RM) was used to replace the experience‐based margin (EM) of 5 mm. Dosimetric differences in the mean lung dose (MLD), V_20_, 0.5‐cm^3^ bronchial dose (BD), 0.4‐cm^3^ spinal cord dose (SD), and 2‐cm^3^ chest wall dose (CD) were compared.

### 2.6 Statistical methods

SPSS 16.0 was used for statistical calculations. Measurement data are presented as $\bar{X}\pm s$. Correlations between two variables were by the Spearman bivariate correlation process. The Shapiro‒Wilk test was used to test the normality of data. Two related samples were compared by the Wilcoxon signed‐rank test. *p* < 0.05 was considered statistically significant.

## 3 RESULTS

### 3.1 Residual errors of the AlignRT 6D‐guided interfraction setup

A total of 59 pretreatment CBCT registration results in 12 patients showed the following residual errors: Vrt_0_, 0.06 ± 0.18 cm; Lng_0_, −0.03 ± 0.19 cm; Lat_0_, 0.02 ± 0.15 cm; Pitch_0_, 0.23 ± 0.7°; Roll_0_, 0.1 ± 0.69°; and Rtn_0_, −0.02 ± 0.79°. The residual errors were small after AlignRT guidance.

### 3.2 Correlation analysis (Figure 3)

The frequency, longest duration, and maximum error in the vertical direction were positively correlated with the treatment duration (*r* = 0.404, 0.353, 0.283, *p* < 0.05, respectively). The frequency of occurrence in the longitudinal direction was positively correlated with age and treatment duration (*r* = 0.376, 0.283, *p* < 0.05, respectively). The maximum error in the longitudinal direction was positively correlated with age (*r* = 0.4, *p* < 0.05). The other variables were not correlated with age or treatment duration. The maximum translation error in the three dimensions during the treatment could exceed the preset 5‐mm PTV margin limit, and the maximum error could even be as high as 14 mm.

### 3.3 Margin analysis

Histograms of the three types of intrafraction error data are shown in Figure 4. The intrafraction error 3 data in the three directions were normally distributed (*p* > 0.05). Table 1 shows the results of these three intrafraction setup errors. For intrafraction error 2, the setup error calculated by the Van Herk correction formula was 2 mm, with a uniform margin. However, for intrafraction error 1, the calculated result in the longitudinal direction needed to be increased by 2 mm to ensure that 90% of the target volume was covered. Intrafraction error 3 showed the largest margin using the Z score analysis method, requiring increases of 3 mm, 5 mm, and 3 mm in the vertical, longitudinal, and lateral directions, respectively. After verifying the AlignRT log files, the results for the three PTV margins in the vertical, longitudinal, and lateral directions were as follows: R_PTV1: 79.7%, 45%, and 75%; R_PTV2: 79.7%, 79.7%, and 75%; and R_PTV3: 92.4%, 93.2%, and 89.8%. When PTV3 was used instead of the experience‐based margin of 5 mm, the PTV volume decreased by 21% on average. The dose to important organs at risk is shown in Figure 5. The MLD, 2‐cm^3^ CD, and lung V_20_ decreased by 0.2 Gy, 2.1 Gy, and 0.5%, respectively (*p* < 0.05), and the 0.5‐cm^3^ BD and 0.4‐cm^3^ SD were slightly reduced (1.6 Gy, *p* = 0.07; 0.3 Gy, *p* = 0.58).

**TABLE 1:** Overview of the three kinds of intrafraction information in the vertical (VRT), longitudinal (LNG), and lateral (LAT) directions as well as the resulting margins.

	Infraction error1 (mm)	Infraction error2 (mm)		Infraction error3 (mm)
Σ VRT	0.7	0.5	*μ*_VRT_	0.3
Σ LNG	1.5	0.8	*μ*_LNG_	0.3
Σ LAT	0.5	0.7	*μ*_LAT_	−0.3
ϕ‐VRT	0.7	0.4	s _VRT_	1.8
ϕ‐LNG	0.9	0.4	s _LNG_	2.9
ϕ‐LAT	0.8	0.4	s _LAT_	1.9
Margins(mm)				
LAT	2	2		3
LNG	4	2		5
VRT	2	2		3

## 4 DISCUSSION

In this study, the six‐dimensional mean residual error of surface‐guided SBRT in lung cancer patients was less than 1 mm and 1°, which is consistent with previous studies showing that SGRT can reduce the interfraction setup error.^30^

Alderliesten et al.^31^, ^32^ used CBCT and SGRT to measure the patient displacement error during SBRT for lung cancer, and they found that patients exceeded the 2 mm threshold during treatment, with the maximum error exceeding 7 mm, Additionally, SGRT monitoring has suggested that the target area may deviate from the original PTV margin range. Purdie et al.^33^ and Shah et al.^34^ demonstrated that the treatment time and the magnitude of patient motion were correlated among composite groups of patients. In this study, we used techniques such as FFF radiation, VMAT, and field angle and multi‐objective optimization to reduce the total number of monitor units and the treatment time.^35^, ^36^, ^37^ However, with the passage of treatment time, the uncertainty of the patient's exposure dose increases and may even alter the patient's respiratory movement. By analyzing the log file, we found that the patient could exceed the movement threshold of 2 mm and 2 s in a single treatment. The frequency, maximum duration, and absolute value of the maximum error in the vertical direction and frequency in the longitudinal direction were positively correlated with the duration of a single treatment. The AlignRT log file recorded the margin range of the PTV exceeding 5 mm during the treatment, and the error even reached 14 mm. This may have been caused by the patient breathing deeply or coughing during the treatment process. Because the error duration threshold is very short and the breathing curve variation is obvious during RPM monitoring, the accelerator beam will be stopped. Therefore, there is no need for great concern over this low‐probability random error in actual treatment. The frequency and absolute value of the maximum error in the longitudinal direction were positively correlated with the age of the patient. This may be because the older the patient is, the poorer their respiratory stability, which increases the random error of the movement in the longitudinal direction caused by breathing. Therefore, the full‐time mode is recommended for older patients to shorten the duration of a single treatment and reduce errors.

**FIGURE 3:**
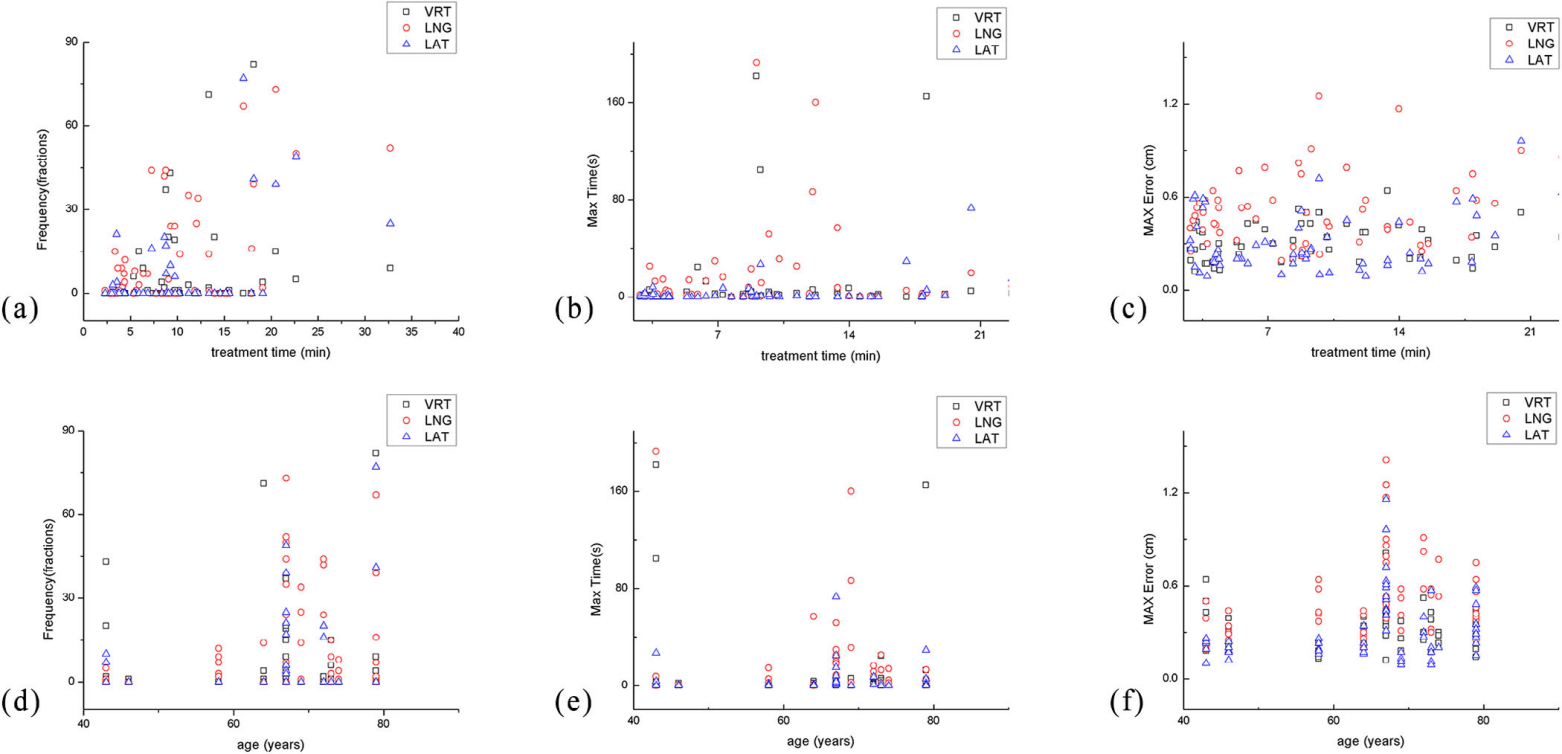
Correlation between frequency, longest duration, and absolute value of the maximum error and patient age and the duration of a single treatment. 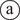. Occurrence frequency and treatment duration. 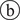. Longest duration and treatment duration. 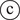. Maximum absolute error and treatment duration. 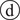. Occurrence frequency and patient age. 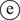. Longest duration and patient age. 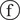. Maximum absolute error and age.

We measured the residual error in three dimensions for CBCT registration and couch movement correction, which was less than 1 mm at our center. In SBRT, CBCT was performed before 6D couch movement based on the registration results before each treatment, which could eliminate the interfraction setup error. The patients underwent strict breathing training before each treatment, and the consistency of their breathing status was verified by kV images. Therefore, the PTV margin range was mainly aimed at the intrafraction error in three directions. The PTV1 and PTV2 calculated using the revised Van Herk formula were small, which may be because the displacement at the end of the treatment or the average displacement cannot represent changes in the patient's position during the entire treatment process. The coverage verification and the number of times the 2‐mm and 2‐s thresholds were exceeded indicated that the tumor coverage rate of the PTV1 and PTV2 margins was low. Therefore, larger margins may be required in the three directions to ensure coverage of the target area. In this study, we used the log file to compute PTV3 by applying Z score analysis, which agrees with the findings of previous studies, in which the sizes in the vertical and lateral directions were reduced from 5 to 3 mm,^38^, ^39^ while a 5 mm margin was still needed in the longitudinal direction. This may be mainly because AlignRT records more cases where the longitudinal direction shift is close to or even more than 5 mm and lasted for an extended duration, as demonstrated in Figure 3 and Figure 5. For displacements caused by either changes in the patient's body position or his or her respiratory movements, the result is insufficient exposure to the edge of the tumor. The movement of the lung tumor is mainly longitudinal, so we believe that it is reasonable to expand the longitudinal margin by 5 mm.

**FIGURE 4:**
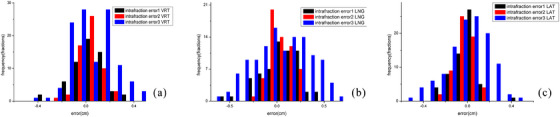
Histograms of the three kinds of intrafraction errors. 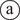. VRT.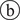. LNG. 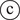. LAT.

**FIGURE 5:**
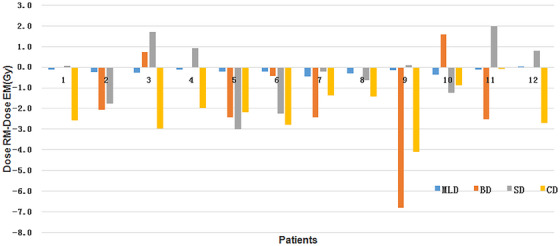
The dose to the rational margin (RM) minus the dose to the experience‐based margin (EM) was used to calculate the mean lung dose (MLD), 0.5‐cm^3^ bronchial dose (BD), 0.4‐cm^3^ spinal cord dose (SD), and 2‐cm^3^ chest wall dose (CD).

Based on the above, we believe that it may be more reasonable to apply the upper and lower limits of the three‐dimensional directional displacement range obtained by analyzing the 95% probability density of the AlignRT monitoring log file for each treatment package as the patient's error within each fraction by applying Z score analysis. Compared with the experience‐based margin, these margins resulted in varying decreases in the dose delivered to vital organs, especially the lung and chest wall. This may be because peripheral lung cancers were more common in this study. Changes in the extent of the margins are relatively sensitive to the lung volume and chest wall distance, whereas the spinal cord and bronchi are in the mediastinal region and are much less affected by changes in these margins.

The sample in this study was small, and the number of treatments administered to each patient was small and not uniform, which may have led to differences in the margin values of the three PTVs. At the same time, to evaluate the coverage of the target area, we simply compared whether the displacement recorded in the log file was within the PTV range, and there was no actual simulated dose coverage.

## 5 CONCLUSION

The AlignRT system offers high setup accuracy, which can reduce the interfraction setup error in patients and allow the displacement error in three directions to be identified during treatment through monitoring. It may be more reasonable to use the upper and lower limits of the 95% probability density interval to replace the displacement at the end of each treatment or the average displacement as the intrafraction error in SBRT to determine the best margin for the PTV in patients with lung cancer. For SBRT with a long single‐treatment time, it is still necessary to have a sufficiently large intrafractional PTV margin to ensure coverage of the target area.

## AUTHOR CONTRIBUTION

Huai‐wen Zhang and Hai‐liang Guo conceived of the presented idea. Hai‐liang Guo and Wei‐wei Wu collected the data of all patients in this study. Huai‐wen Zhang and Hai‐liang Guo took the lead in writing the manuscript. All authors provided critical feedback and helped shape the research, analysis, and manuscript.

## CONFLICT OF INTEREST STATEMENT

There are no conflicts of interest to declare.

## ETHICS APPROVAL AND CONSENT TO PARTICIPATE

According to the ethical guide‐lines of the Helsinki Declaration and was approved by the institutional review board of the First Affiliated Hospital of Gannan Medical University. Written informed consents were obtained from all patients prior to treatment.

## CONSENT FOR PUBLICATION

Consent for publication is not applicable in this study, because there is not any individual person's data.

## Data Availability

All data generated and analyzed during this study are included in this published article. The data that support the findings of this study are available from the corresponding author, Huai‐wen Zhang, upon reasonable request.
